# Effect of phthalocyanine oral and nasal antiseptic solutions on the infectivity of SARS-CoV-2 in patients with COVID-19: a randomized controlled trial

**DOI:** 10.3205/000321

**Published:** 2023-06-23

**Authors:** Andréa Name Colado Simão, Nicole Perugini Stadtlober, Audrey Alesandra Stinghen Garcia Lonni, Luiza Mara Venâncio, Guilherme Lerner Trigo, Pedro Luis Candido de Souza Cassela, Thais Mastellini Sanches Silva, Maria De Fátima Oliveira Hirth Ruiz, Marcell Alysson Batisti Lozovoy, Zuleica Naomi Tano, Bernardo da Fonseca Orcina, Fabiano Vieira Vilhena, Paulo Sérgio da Silva Santos

**Affiliations:** 1 Research Laboratory in Applied Immunology, State University of Londrina, Brazil; 2 Department of Pharmaceutical Sciences, State University of Londrina, Brazil; 3 Department of Surgery, Stomatology, Pathology and Radiology, Bauru School of Dentistry, University of São Paulo, Bauru, Brazil; 4 TRIALS – Oral Health & Technologies, Bauru, Brazil

**Keywords:** COVID-19, SARS-CoV-2 infection, infectivity, phthalocyanine, mouthwashes

## Abstract

**Background:**

In individuals with coronavirus disease (COVID-19), the severe acute respiratory syndrome coronavirus 2 (SARS-CoV-2) viral load (VL) plays an important role in infectivity.

**Objectives:**

This study aimed to evaluate the reduction in the VL and infectivity induced by phthalocyanine mouthwash and nasal spray in patients with COVID-19.

**Methods:**

Patients with mild COVID-19 were recruited to participate in a triple-blinded randomized controlled trial. Participants were assigned to one of three groups: Group 1, non-active mouthwash and saline nasal spray (SNS); Group 2, phthalocyanine mouthwash and SNS; and Group 3 phthalocyanine mouthwash and phthalocyanine nasal spray. VL was assessed in nasopharyngeal and oropharyngeal swabs collected at the time of clinical diagnosis at baseline as well as 24 and 72 hours after starting the rinsing protocols.

**Findings:**

Forty-six participants were included in the analysis: 15, 16, and 15 in Groups 1, 2, and 3, respectively. After 72 hours, the reduction in VL was significantly higher in Group 3 (mean cycle threshold (Ct) decrease: 11.21) than in Group 1 (mean Ct decrease: 5.53). Additionally, only the mean VL in Group 3 was reduced to a non-contagious level after 72 hours.

**Main conclusions:**

Use of phthalocyanine mouthwash and nasal spray is effective at reducing SARS-CoV-2 infectivity.

## Zusammenfassung

**Hintergrund:**

Bei Personen mit Coronavirus-Erkrankung (COVID-19) spielt die Viruslast (VL) des schweren akuten respiratorischen Syndroms Coronavirus 2 (SARS-CoV-2) eine wichtige Rolle für die Infektiosität.

**Zielsetzungen:**

Ziel dieser Studie war es, die Verringerung der VL und der Infektiosität durch Phthalocyanin-Mundwasser und -Nasenspray bei Patienten mit COVID-19 zu untersuchen.

**Methoden:**

Patienten mit leichter COVID-19-Erkrankung wurden für die Teilnahme an einer dreifachblinden, randomisierten und kontrollierten Studie rekrutiert. Die Teilnehmer wurden einer von drei Gruppen zugeteilt: Gruppe 1: nicht-aktive Mundspülung und Kochsalz-Nasenspray; Gruppe 2: Phthalocyanin-Mundspülung und Kochsalz-Nasenspray und Gruppe 3: Phthalocyanin-Mundspülung und Phthalocyanin-Nasenspray. VL wurde in nasopharyngealen und oropharyngealen Abstrichen untersucht, die zum Zeitpunkt der klinischen Diagnose zu Studienbeginn sowie 24 und 72 Stunden nach Beginn der Spülprotokolle entnommen wurden.

**Ergebnisse:**

In die Analyse wurden 46 Teilnehmer einbezogen: 15 in Gruppe 1, 16 in Gruppe 2 und 15 in Gruppe 3. Nach 72 Stunden war die Verringerung der VL in Gruppe 3 signifikant höher (mittlere Abnahme der Zyklusschwelle: 11,21) als in Gruppe 1 (mittlere Abnahme der Zyklusschwelle: 5,53). Außerdem war nur die mittlere VL in Gruppe 3 nach 72 Stunden auf ein nicht-ansteckendes Niveau gesunken.

**Wichtigste Schlussfolgerungen:**

Die Verwendung von Phthalocyanin-Mundspülungen und -Nasensprays ist wirksam bei der Verringerung der SARS-CoV-2-Infektiosität.

## Introduction

The coronavirus disease (COVID-19) pandemic began in December 2019 in China and spread worldwide, causing serious consequences and prompting a race to search for drugs to treat and prevent the disease. The nasal and oral cavities contain a large number of angiotensin-converting enzyme [1] (ACE2) and TMPRSS [2] receptors, which serve as binding sites by which severe acute respiratory syndrome coronavirus 2 (SARS-CoV-2) enters cells. Once infected, person-to-person transmission can occur through human saliva, respiratory droplets, potentially through direct contact and fomites, and by speaking, coughing, sneezing, or breathing, and the respiratory droplets formed may contain a high viral load (VL) [3], [4]. The SARS-CoV-2 VL plays an important role in infectivity and transmission, in addition to affecting the body of infected individuals [5], [6], [7]. Faced with this situation, preventive measures are necessary, including paying attention to simple measures to promote oral and nasal hygiene. Antiviral solutions ranging from simple universal saline solutions to novel compounds have been proposed for mechanical as well as chemical oral and nasal antisepsis [8], [9], [10]. Recent research has shown that an iron phthalocyanine derivative has antiviral and anti-inflammatory properties. Without the aid of light or any other external source, this self-activated phthalocyanine derivative relies on self-activation and localized generation of reactive oxygen. As an adjuvant therapy for the prevention and treatment of patients with SARS-CoV-2 infection, an active mouthwash containing an iron phthalocyanine derivative has demonstrated encouraging outcomes [11], [12], [13]. This study aimed to evaluate the reduction in the VL and infectivity induced by nasal and oral rinsing with phthalocyanine in patients with COVID-19.

## Patients and methods

### Study design

Patients were recruited from the COVID-19 outpatient clinic of the University Hospital of Londrina-Paraná, Brazil, from November 1, 2020, to February 1, 2021. The study was a prospective randomized triple-blinded randomized controlled trial. Participants were instructed to use nasal and oral rinsing protocols according to the following groups: Group 1, non-active mouthwash and saline nasal spray (SNS); Group 2, phthalocyanine mouthwash and SNS; and Group 3, phthalocyanine mouthwash and phthalocyanine nasal spray. They were instructed to gargle for 30 sec and rinse for 30 sec with 5 ml of the mouthwash solution and to spray two pumps of the nasal spray into each nostril five times a day for three consecutive days. Participants’ VLs were assessed using reverse-transcription polymerase chain reaction (RT-PCR) on the first and third days of using the mouthwash and nasal spray. Each participant’s adherence to the rinsing protocols was recorded.

The inclusion criteria were patients aged 18 to 80 years who tested positive for SARS-CoV-2 and were diagnosed within 7 days of symptom onset. The exclusion criteria included patients with cycle threshold (Ct) values greater than 31 and patients who had contraindications to using mouthwash or nasal spray for medical reasons or because of an inability to gargle and spit. All participants received standard care (antibiotics, corticosteroids, and anticoagulants) according to the World Health Organization standard treatment guidelines [14] plus one of the three mouthwash and nasal spray interventions. The phthalocyanine mouthwash had the same formula (color, flavor, and other ingredients) as a non-active mouthwash, except for the presence or absence of the active ingredient, and both were produced by Rabbit Corp, Brazil. A private dispensing pharmacy in Brazil produced the phthalocyanine nasal solution. Both solutions (phthalocyanine and SNS) were bottled in exactly the same type of container with a pump valve.

### Randomization and masking

Sample randomization was performed as follows: the mouthwash bottles and nasal sprays were placed in consecutively numbered closed packages. An Excel (Microsoft, Redmond, WA, US) database was created from these numbered packages and used for randomization. After randomization, packages with mouthwash bottles, oral care kits (dental floss, toothbrush, and fluoride toothpaste, Rabbit Corp, Brazil), and nasal sprays were delivered to the participants.

### RNA extraction and quantitative PCR for SARS-CoV-2

Viral RNA was extracted from 100 µL of nasopharyngeal or oropharyngeal swab, using the automated extractor, EXTRACTA 32 (Loccus, Cotia, Brazil), and magnetic bead extraction kits (MVXA-P016 FAST), and following the manufacturer’s instructions (Loccus, Cotia, Brazil). A negative extraction control (UltraPure DNase/RNase-Free Distilled Water, Thermo Fisher Scientific, Waltham, MA, USA) was added to each extraction run.

The diagnosis of SARS-CoV-2 infection and monitoring of VL were performed by real-time reverse transcription-polymerase chain reaction (RT-PCR) using fluorescent probes (Taqman) [15]. Nucleic acids were extracted from 120 µL of nasopharyngeal or oropharyngeal swab collected by an experienced nurse using the EXTRACTA Kit FAST-DNA and viral RNA and following the manufacturer’s instructions (Loccus, São Paulo, Brazil). The purified nucleic acid was reverse-transcribed into cDNA and amplified using the TaqPath COVID-19 CE-IVD RT-PCR Kit (Applied Biosystems) and the QuantStudio 6 Flex Real-Time PCR System, according to the manufacturer’s instructions. The RT-PCR result was considered positive when two or more genes of SARS-CoV-2 (ORF1ab, N, and S) were present with Ct values less than 37. The mean Ct values of the three genes were used for the statistical analysis. Real-time RT-PCR Ct is a semi-quantitative method of estimating the VL. The Ct value represents the number of cycles required to identify the target gene [16], and is inversely related to the VL. The VL was classified as high, intermediate, or low, according to whether the Ct value was <25, 25–30, or >30, respectively [5]. According to Jaafar et al. [7], infectivity, as defined by growth in cell culture, can be considered high when the RT-PCR Ct value is less than 25. In addition, approximately 85% of cultures have been shown to be negative if the Ct value is greater than 33. Thus, we considered samples with Ct values greater than 33 to have low infectivity.

### Statistical analysis

Initially, descriptive and exploratory analyses of all data were performed. Data were summarized as frequencies and percentages for categorical variables and as means and standard deviations or medians and interquartile ranges for continuous variables. Then, the three groups were compared using Fisher’s exact test for categorical variables and generalized linear models for continuous variables (age and onset of symptoms). The Ct values met the assumptions for parametric analysis and were analyzed using generalized linear models for repeated measures. They were also analyzed for variations in the Ct value in relation to the baseline by one-way analysis of variance and Tukey’s test. Finally, associations between groups and a classification of infectivity (high, intermediate, and low) were analyzed based on Ct values using Fisher’s exact test. All analyses were performed using the R Core Team program (R Foundation for Statistical Computing, Vienna, Austria) with a significance level of 5%. All tests were based on the null hypothesis of the three interventions having an equivalent effect on the Ct value.

## Results

**Figure 1:**
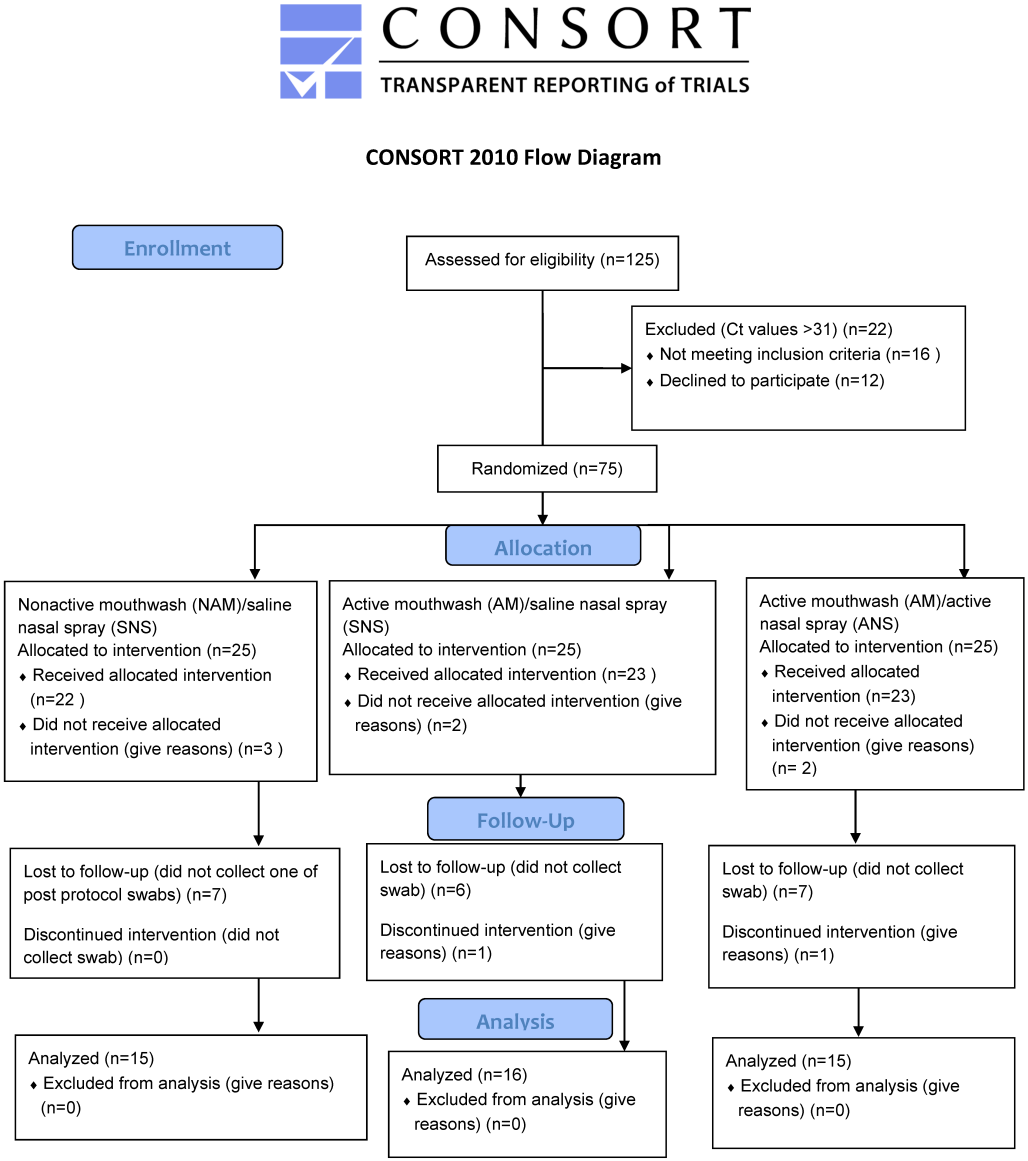
CONSORT flow diagram

A total of seventy-five patients with mild COVID-19 were recruited for the study (Figure 1 (Fig. 1)). Twenty-five patients were assigned to each group. After removing seven patients who did not satisfy the inclusion criteria, there were 22, 23, and 23 patients in Groups 1, 2, and 3, respectively (n=68). After excluding patients who declined to participate or who discontinued the intervention, there were 15, 16, and 15 participants in Groups 1, 2, and 3, respectively, (n=46) who completed the intervention and were included in the analysis.

As shown in Table 1 (Tab. 1), there was no significant difference between the groups in terms of age and time since symptom onset. The patients’ ages ranged from 20 to 66 years, with means of 33.9 years in Group 1 (use of non-active mouthwash and SNS), 36.6 years in Group 2 (use of mouthwash containing phthalocyanine and SNS), and 41.3 years in Group 3 (use of phthalocyanine mouthwash and phthalocyanine nasal spray). There was also no difference between the groups in terms of sex distribution, presence of obesity, hypertension, diabetes, or other comorbidities.

The Ct values decreased significantly in all three groups (Table 2 (Tab. 2)). After 72 hours, the decrease in the Ct was greatest in Group 3 (11.21) and lowest in Group 1 (5.53).

**Figure 2:**
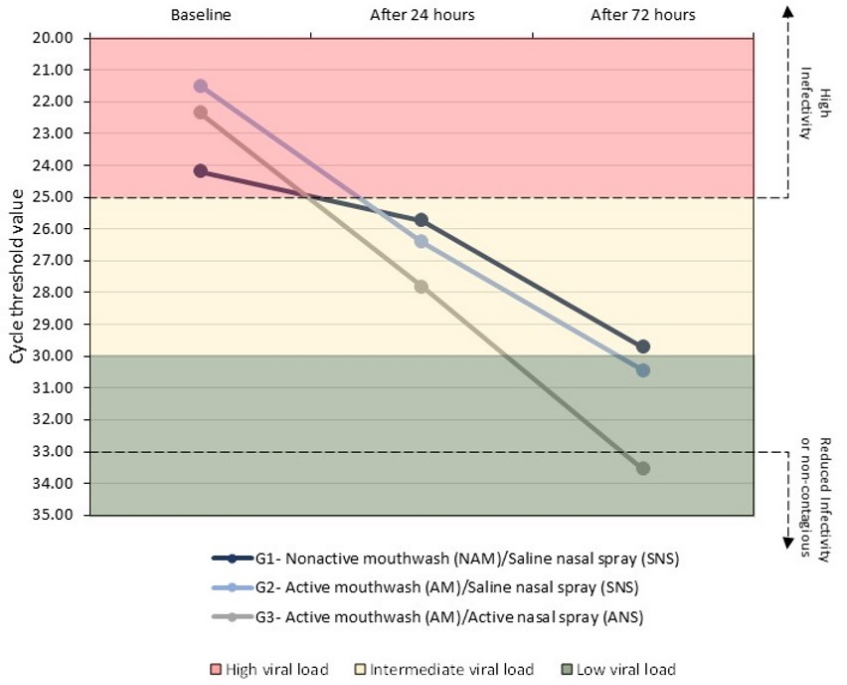
Qualitative data of mean VL and infectivity estimated by the Ct value as a function of group and time

Figure 2 (Fig. 2) shows the mean Ct values of the three groups over time, according to the degree of infectivity and the VL. All three groups had a high VL at baseline. Seventy-two hours after starting the rinsing protocols, Group 1 had an intermediate VL while Groups 2 and 3 had a low VL. Only Group 3 had a mean Ct value showing reduced infectivity on day 3.

## Discussion

In the present study, all three groups experienced a decrease in Ct values over time, indicating a reduction in the VL. These results are in accordance with previous reports that gargling and rinsing with mouthwash as well as rinsing the nasal cavity are associated with a reduction in the SARS-CoV-2 VL [9], [17].

Considering that the highest VL can be found in the first week after symptom onset [18], a rapid reduction in the VL as soon as the patient is diagnosed with COVID-19 is important. SARS-CoV-2 VL is associated with markers of inflammation and disease severity [19]. In the presence of inflammation and severe disease, the WHO COVID-19 drug protocol recommends the use of additional therapies to reduce the VL, and a reduction in the VL can contribute to a better clinical outcome [13]. In this study, there was a threefold greater reduction in the VL in the first 24 hours and a greater than 1.5-fold greater reduction after 72 hours in the groups that used the phthalocyanine mouthwash (Groups 2 and 3) compared to the group that used non-active mouthwash (Group 1). In addition, only in the groups that used the phthalocyanine mouthwash (Groups 2 and 3) had low VLs (Ct>30) 72 hours after starting the rinsing protocols. The use of phthalocyanine solutions was shown to have a clinically important effect. Previous laboratory studies have shown greater than 90% reduction in the SARS-CoV-2 VL when samples were exposed to phthalocyanine [13], [20]. In addition, clinical improvement using the adjuvant phthalocyanine gargling and rinsing mouthwash protocol was achieved, leading to a favorable asymptomatic outcome after a few days of use for both hospitalized and non-hospitalized COVID-19 patients [11], [12], [13].

This study has some limitations. The sample size was limited. Furthermore, Ct values are semi-quantitative and the RT-PCR evaluation only indicates the presence or absence of virus fragments. More precise results could be obtained if the evaluation measured the infective capacity of viable viruses after using the rinsing protocols. 

In the present study, we adopted the measure of infectivity as previously described by Jaafar et al. [7], based on a study in which more than 3,700 samples were assessed for viral viability. The study results showed that samples with Ct values lower than 25 had high infectivity and samples with Ct values of 33 or more had reduced infectivity. Lowering infectivity reduces both the individual risk of clinical worsening, and the risk of SARS-CoV-2 transmission to other individuals. In our study, only the group that used both phthalocyanine mouthwash and phthalocyanine nasal spray (Group 3) showed reduced infectivity 72 hours after beginning the protocol.

## Conclusion

Nasal and oral rinsing with phthalocyanine antiseptic solutions can reduce the SARS-CoV-2 VL and reduce infectivity to low or non-contagious levels after 3 days of use. Further studies with larger sample sizes are required to confirm these results.

## Notes

### Acknowledgements

We thank Dr. Lucas Marques da Costa Alves for his support regarding the topic of infectious diseases.

### Patient consent

All participants provided written informed consent.

### Ethics statement

The study protocol was approved by the Research Ethics Committees of the State University of Londrina, Paraná, Brazil (CAAE 37277420.0.0000.5231), and the followed procedures were in accordance with the Helsinki Declaration of 1975, as revised in 1983.

The study was registered in the Brazilian Registry of Clinical Trials (ReBEC) (RBR-8tygcz7) and complied with the Consolidated Standards of Reporting Trials (CONSORT) 2010 checklist.

### Authors’ contributions

- Study design, article writing, data analysis, and final review: ANCS, FVV, BFO, PSSS
- Study design: AASGL, ZNT
- Data collection: LMV, NPS, GLT, PLCSC, MFOHR, TMSS, MFOHR
- Data analysis: MABL

### Conflict of interests

Dr. Vilhena has a patent classification pending.

Prof. Dr. da Silva Santos reports grants from CNPq process nº 309525/2018-7.

The other authors declare that they have no competing interests.

## Figures and Tables

**Table 1:**
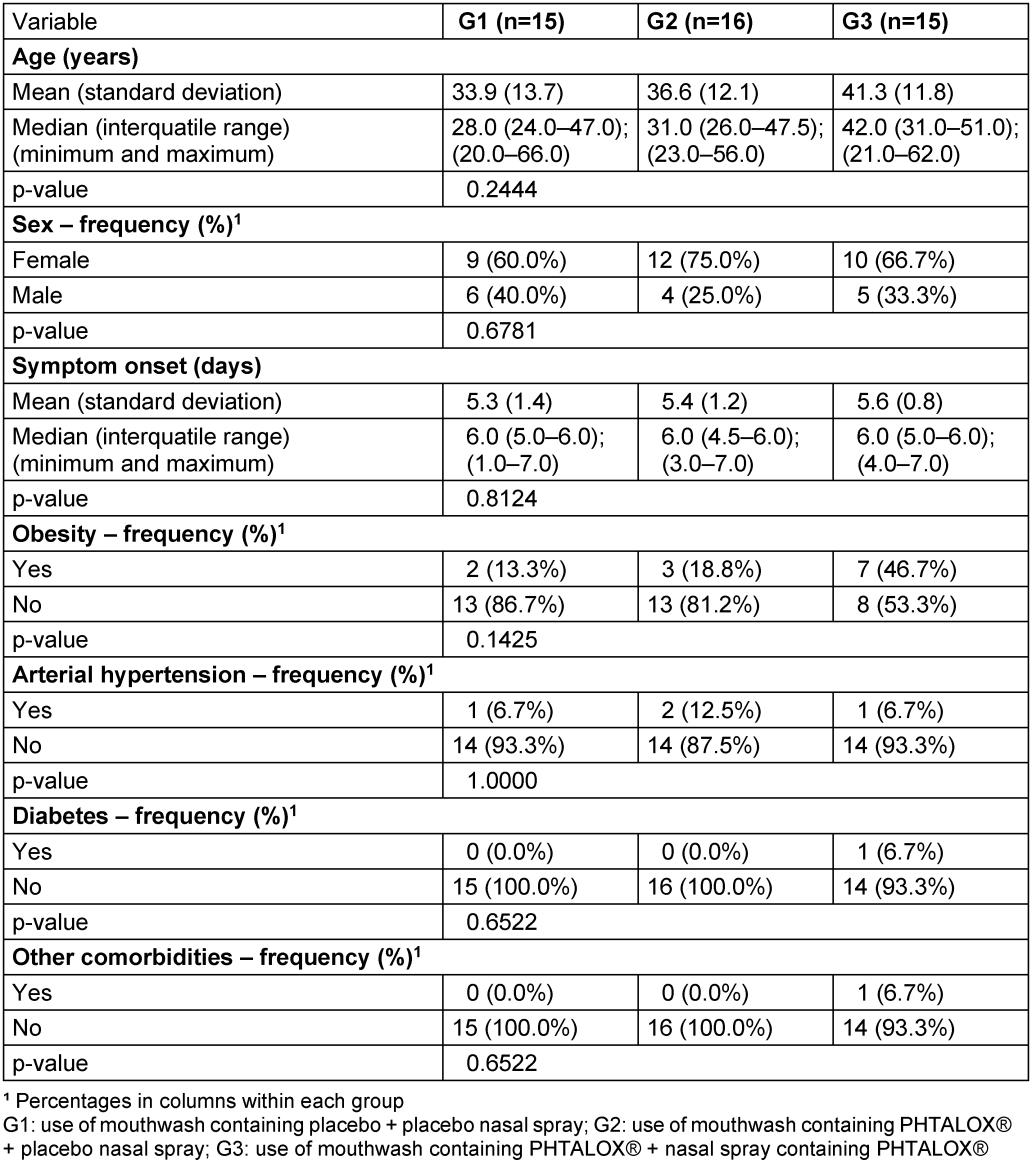
Comparisons between groups (G1–3) regarding patient characteristics

**Table 2:**
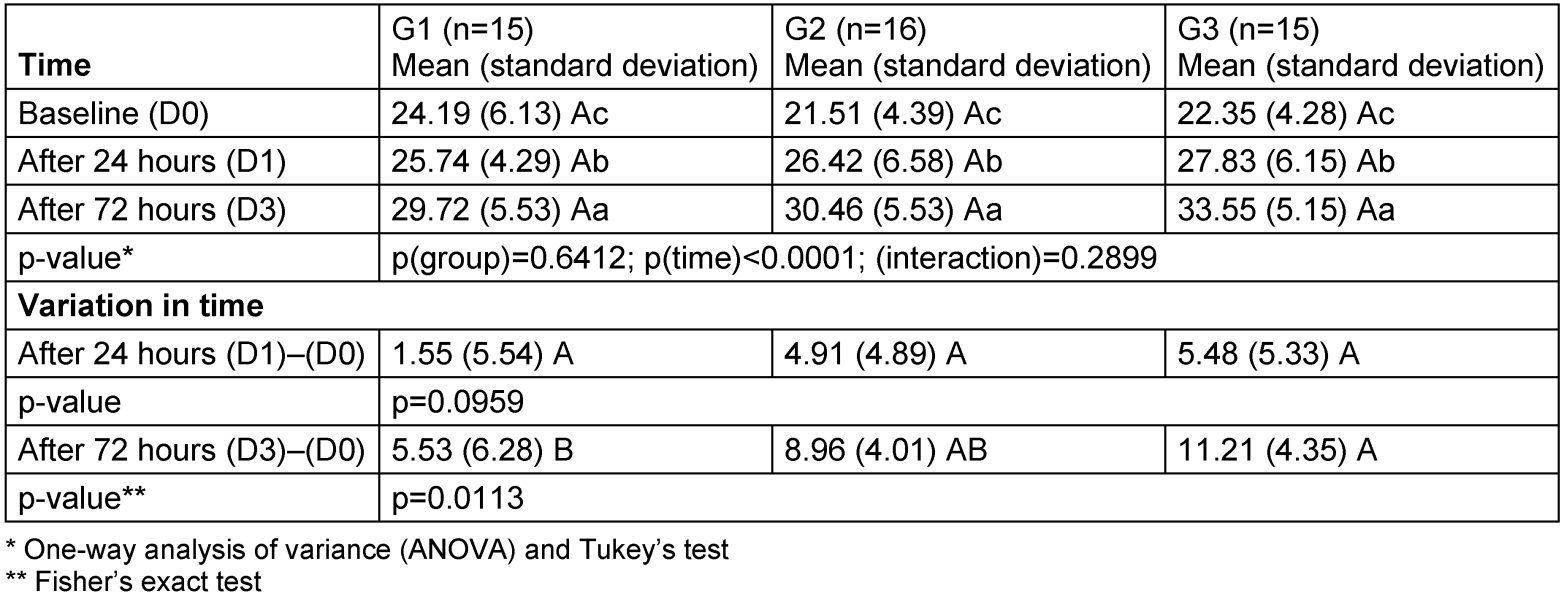
Mean (standard deviation) of the VL estimated by the Ct value obtained in the PCR reaction as a function of the group and time. Different letters (upper case comparing horizontally between groups and lowercase comparing vertically between times) indicate statistically significant differences. Group 1 (G1): use of mouthwash containing placebo + placebo nasal spray; Group 2 (G2): use of mouthwash containing phthalocyanine + placebo nasal spray; Group 3 (G3): use of mouthwash containing phthalocyanine + nasal spray containing phthalocyanine
